# Bullying and cyberbullying. A high risk, in boys and girls, of superficial learning, poor planning and academic procrastination

**DOI:** 10.3389/fpsyg.2025.1567523

**Published:** 2025-07-10

**Authors:** Alba Rusillo-Magdaleno, Manuel J. de la Torre-Cruz, Teresa Martínez-Redecillas, Alberto Ruiz-Ariza

**Affiliations:** ^1^Faculty of Humanities and Educational Sciences, Department of Didactics of Musical, Plastic and Corporal Expression, University of Jaén, Jaén, Spain; ^2^Faculty of Humanities and Educational Sciences, Department of Psychology, University of Jaén, Jaén, Spain; ^3^Faculty of Philosophy and Letters, Department of English and German Philology, University of Granada, Granada, Spain

**Keywords:** aggression, victimization, bullying, cyberbullying, teaching, learning, schoolchildren, decision-making

## Abstract

The aim of the present study was to analyse the association of bullying and cyberbullying with deep learning, superficial learning, planning and decision making, as well as school procrastination. A total of 1,263 Spanish schoolchildren (51.39% girls) aged 10–16 years (13.23 ± 1.77) participated. The association between variables and the analysis of exposure risk was performed by analysis of covariance (ANCOVA) and binary logistic regression, respectively. All analyses were conducted separately for boys and girls and adjusted for age, body mass index, mother's education and average weekly physical activity. Results showed that girls who were victims of bullying and cyberbullying had significantly higher procrastination toward class tasks (7 and 16%, respectively). In addition, cyberbullying victims acquire more superficial learning (5.28%). In general, victims of bullying have almost twice the risk of having higher values of superficial learning and procrastination than non-victims. This risk is multiplied by 3 and 4, respectively, in the case of cyberbullying victims. On the other hand, bullying aggressors were also found to have high superficial learning (7.34%) and higher procrastination (17.45%). In the case of cyberbullying, aggressors also had more superficial learning (boys = 13.38% and girls = 9.56%), worse values in planning and decision making (boys = 3.82% and girls = 3.3%) and more procrastination (boys = 16.81% and girls = 20.48%). In both sexes, the risk of exposure to aggression toward the above variables is multiplied by 8, 2, and 10, respectively. All these findings reveal that bullying and cyberbullying can affect young people in key learning variables, beyond those of physical, psychological or socio-emotional aspects already known. Immediate and systematic actions are needed to monitor and prevent bullying and cyberbullying inside and outside the school context, creating safe spaces and providing counseling for both victims and aggressors.

## 1 Introduction

Bullying has been defined as a manifestation of mistreatment between students, characterized by acts of physical or mental violence and sustained over time (Nain et al., 2023). This behavior can be perpetrated by one or several individuals and is directed toward another subject or group that is in a situation of inability to defend itself (Nain et al., 2023; Olweus, 1978). This type of mistreatment generates episodes of aggression and victimization that provoke a hostile environment in the classroom and lead to consequences such as anxiety crises, isolation and a decrease in interest in learning (Cerezo, 2002; Rigby, 2000). It has been found that 32% of the adolescent population has experienced bullying by their peers at least once a month (UNESCO, 2019), however, these values could increase as bullying is adopting new forms such as cyberbullying (Makarova and Makarova, 2023).

Cyberbullying refers to the use of digital media such as social networks, messaging apps and websites with the intent to intentionally and repeatedly harass, threaten, humiliate or harm a person (Livazovi and Ham, 2019; Martínez-Soto and Ibabe, 2024). This modern bullying procedure is distinguished by its ability to transcend physical barriers as it allows aggressors to attack their victims anytime, anywhere (Ramadan, 2023). Characteristics of cyberbullying include spreading rumors, publishing personal information without consent, and creating fake profiles to damage someone's reputation (Palade and Pascal, 2023). This form of bullying has experienced a considerable rise internationally and has attracted significant attention from researchers in the last 5 years (Kumari et al., 2020). A relevant feature that differentiates this form of bullying from traditional bullying is the anonymity offered by the platforms as they allow bullies to act without fear of retaliation (Boichuk et al., 2023) and traditional bullying can occur in physical environments and cyberbullying through digital platforms (Ramadan, 2023). With regard to bullying roles, it has been observed that victims tend to manifest passive behavior, while bullies can be classified into two categories: active and passive (Laninga-Wijnen et al., 2023; Revuelta Domínguez et al., 2023; Santoyo and Frías, 2014). Bullies, unlike victims, demonstrate ostensible self-confidence (Laninga-Wijnen et al., 2023; Revuelta Domínguez et al., 2023; Santoyo and Frías, 2014), tend to lead groups (Santoyo and Frías, 2014) and report experiencing feelings of happiness or anger during the act of bullying (Pedditzi et al., 2022).

Despite the above differences, bullying and cyberbullying represent a common problem among students in educational institutions (Javed et al., 2023). This phenomenon has reported negative effects on physical (physical violence) (Tintori et al., 2021), psychological (verbal violence or insults) (Shi et al., 2024) or socio-emotional (isolation) aspects (Alotaibi, 2019; Fullchange and Furlong, 2016), but there are still few studies that analyse the direct effect on key aspects for comprehensive youth development such as learning. Some variables that have already begun to be studied in this line are the negative effects on the attitude toward education (de Benítez-Sillero et al., 2021), poor quality learning (Graham, 2023), or the inadequate study strategies it provokes in students involved in bullying (Aparisi et al., 2021).

In this context, learning-related variables have been assessed by monitoring the acquisition of learning through methods that differentiate between deep learning and superficial learning (Matton and Svensson, 1979). Deep learning is defined as an educational process by which students achieve comprehensive understanding by implementing cognitive strategies of analysis and inference (Çetín and Demirtaş, 2022) and is characterized by providing intrinsic motivation (Badawi, 2023). In contrast, superficial learning is identified by the student's inability to relate new knowledge to previously acquired information, favoring a passive approach focused on memorization and the application of basic cognitive strategies (Santrock, 2006). This extrinsically motivated category of knowledge acquisition is distinguished by a constant preoccupation with potential school failure (Entwistle and Ramsden, 1983).

On the other hand, other variables that determine effectiveness toward the acquisition of competences in students are planning and decision making toward classroom tasks. Planning is defined as the set of steps to be taken to achieve a specific goal, which involve the organization and recognition of the task (Baggetta and Alexander, 2016). Students with low perceived stress, high self-esteem and lower frustration have been found to have high levels of planning (Ibáñez et al., 2018; Valiente-Barroso et al., 2021). Moreover, bullying experiences may result in poor learning planning (McNaughton et al., 2023). Another relevant aspect is related to decision-making, characterized by following a complex process involving phases of problem identification and analysis, evaluation of personal decisions, and exploration of alternative solutions (Oliva et al., 2011; Pardos and María González Ruiz, 2018). Despite the above, planning, decision-making and task completion may be postponed for various reasons such as procrastination. Procrastination is described as the tendency of students to intentionally delay relevant tasks or decisions, opting instead for more pleasurable activities (Olleras et al., 2022). The main characteristic resulting from procrastination behavior is the heavy workload at the last minute (Kuftyak, 2022). As a result, the perpetuation of the procrastination cycle can lead to elevated levels of stress and anxiety (Mohammadi Bytamar et al., 2020), low self-esteem, lack of self-regulation and intrinsic motivation (Tan and Pang, 2023) and consequently low learning efficacy (Xu, 2023; Xu et al., 2023).

All of the above allows us to identify a complex relationship between bullying and learning variables that may be, in turn, affected by many other covariates. Specifically, it has been observed that bullying tends to occur mainly between students of the same sex (Bonet-Morro et al., 2022). In addition, bullied girls are at a higher risk of poor academic performance compared to boys (Riffle et al., 2021) and tend to seek outside help, while boys tend to respond at the time or later (Bonet-Morro et al., 2022). Another relevant variable, in the context of learning and bullying dynamics, is the inclusion of the mother's educational level due to its influence on attitudes, knowledge and practices related to parenting and child rearing (Masapanta-Andrade and Alvear-Arévalo, 2023; Montes Quiroz et al., 2023). Recent studies have reported the importance of analyzing the mother's educational level to control for effects and to obtain more accurate and reliable results in research related to adolescents' academic development and psychosocial wellbeing (Baharvand et al., 2021).

Finally, there are other variables that may affect many young people and also have a complex relationship with bullying (García-Hermoso et al., 2020; Lavay, 2015; Méndez et al., 2019). On the one hand, students who practiced physical activity at least four times a week show higher values of aggressiveness compared to students who practiced less frequently (Méndez et al., 2019). On the other hand, not meeting the recommended weekly physical activity guidelines seems to be associated with 14% more bullying victimization (García-Hermoso et al., 2020). In the educational context, bullying was one of the main reasons why students dropped out of Physical Education classes, with 11.1% of students experiencing physical bullying, 13.6% verbal bullying and 12.8% social bullying (Lavay, 2015). The relationship between physical activity, aggressiveness and victimization can be explained by the different roles that students assume in physical education. Those who practice more physical activity tend to occupy positions of dominance in competitive contexts, which may favor aggressive behaviors. On the other hand, those who are less active present lower motor competence and physical self-esteem, which makes them more vulnerable to exclusion and bullying. Thus, the level of physical practice is associated with both aggression and victimization, although by different mechanisms conditioned by the social and pedagogical context of the classroom (Rusillo-Magdaleno et al., 2024). Moreover, a high body mass index (BMI) is present in a large proportion of bullying-related cases (Bacchini et al., 2015; Cheng et al., 2022; Lian et al., 2018). Overweight young people were 26% more likely to experience bullying victimization than those of normal weight (Ganapathy et al., 2019; Lee et al., 2018; Pérez-de Corcho et al., 2023). In the case of cyberbullying, youth obesity is also present in 17.2% of cases (Sergentanis et al., 2021). However, other studies found no significant associations between obesity and cyberbullying (Lee et al., 2018; Wang et al., 2010). Age is also a possible confounding variable given its relevance in previous studies, where cognitive and emotional maturity have been found to significantly influence how individuals learn and interact with their environment (Zaatari and El Maalouf, 2022; Urruticoechea et al., 2021). Academic performance, mental health and IQ have also been found to be significantly associated with mothers' educational attainment (Baharvand et al., 2021).

The present research adopts an innovative approach by analyzing not only the repercussions of bullying on the victims, but also its the aggressors themselves. Likewise, the influence of bullying on academic performance and the learning process remains an area of study with limited quantification. To date, no precise metrics have been developed to assess the level of risk to which students involved in bullying situations are exposed, which makes it difficult to design preventive strategies based on empirical and quantifiable data. Based on the above, the aim of this study was to analyze the association between victims and perpetrators of bullying and cyberbullying with deep learning, superficial learning, planning and decision-making and procrastination in schoolchildren and adolescents of both sexes in the Spanish population, after adjusting for age, BMI, mother's level of education and average AFMV. The present study hypothesized that both young victims and perpetrators of bullying and cyberbullying have a poorer quality of learning and present a higher risk of superficial learning, low planning, difficulties in decision making and school procrastination than those who are not involved in bullying and cyberbullying situations.

## 2 Methods

### 2.1 Participants

A total of 1,263 primary and secondary school students aged 10–16 years (13.23 ± 1.76 years, 51.38% girls) participated in the present cross-sectional quantitative study. Data collection took place between February and May 2023. Students from seven schools in the autonomous community of Andalusia (Spain) were surveyed. Schools were selected by convenience and participants were selected randomly and proportionally to the total number of each class group. Anthropometric and sociodemographic characteristics are detailed in Table 1.

**Table 1:** Biometric and sociodemographic characteristics, bullying/cyberbullying (behaviors described during the last 2 months), learning and confounding variables in adolescents, segmented by sex.

**Variables**	**All (*****n*** = **1,263)**		**Boy (*****n*** = **614)**		**Girls (*****n*** = **649)**		***p***
	**Mean/** ***N***	**SD/%**	**Mean/** ***N***	**SD/%**	**Mean/** ***N***	**SD/%**	
Age (years)	13.23	1.77	13.22	1.81	13.25	1.72	0.801
Weight (kg)	52.38	13.54	54.79	15.04	50.09	11.5	< 0.001
Size (m)	1.59	0.11	1.61	0.13	1.57	0.08	< 0.001
Body mass index (kg/m^2^)	20.52	4.02	20.81	3.96	20.24	4.06	0.012
**Mother's school level (%)**							
No studies	4.80%		4.60%		4.90%		0.02
Elementary studies	10.50%		10.70%		10.20%		
Secondary studies	14.40%		11.40%		17.30%		
Professional training	13.60%		13.70%		13.60%		
University studies	35.40%		32.60%		38.10%		
N/C	20.40%		25.10%		16%		
Mean MVPA	4.01	1.76	4.3	1.81	3.73	1.67	< 0.001
**Bullying victimization**							
Never	192	15.2	110	17.9	82	12.6	0.058
Once or twice	668	52.9	309	50.3	359	55.3	
Once or twice/month	301	23.8	140	22.8	161	24.8	
Once/week	82	6.5	45	7.3	37	5.7	
More than once/week	20	1.6	10	1.6	10	1.5	
**Bullying aggression**							
Never	352	27.9	158	25.7	194	29.9	0.02
Once or twice	716	56.7	344	56	372	57.3	
Once or twice/month	153	12.1	90	14.7	63	9.7	
Once/week	34	2.7	20	3.3	14	2.2	
More than once/week	8	0.6	2	0.3	6	0.9	
**Cyberbullying victimization**							
Never	556	44	296	48.2	260	40.1	0.025
Once or twice	625	49.5	282	45.9	343	52.9	
Once or twice/month	61	4.8	26	4.2	35	5.4	
Once/week	19	1.5	8	1.3	11	1.7	
More than once/week	2	0.2	2	0.3	0	0	
**Cyberbullying aggression**							
Never	718	56.8	346	56.4	372	57.3	0.389
Once or twice	485	38.4	235	38.3	250	38.5	
Once or twice/month	37	2.9	23	3.7	14	2.2	
Once/week	23	1.8	10	1.6	13	2	
More than once/week	0	0	0	0	0	0	
Deep learning	2.89	0.72	2.83	0.74	2.95	0.7	0.004
Superficial learning	2.73	0.76	2.84	0.76	2.62	0,.4	< 0.001
Planning and decision making	5.46	0.97	5.35	1.04	5.56	0.88	< 0.001
Procrastination	2.39	0.98	2.49	1.01	2.28	0.95	< 0.001

### 2.2 Dependent variables

#### 2.2.1 Attitudes toward study

The revised version of the “Revised study process questionnaire” (R-SPQ-2F) (Biggs et al., 2001) was used to measure attitudes toward study. This version included 20 items and was structured in two dimensions. Factor 1: Superficial learning (10 items, e.g., < < I learn some things by heart and repeat them, over and over again, even though I do not understand them>>) and Factor 2: Deep learning (10 items, e.g., < < I ask myself questions about subject topics to see if I have understood them clearly>>). Responses are scored on a Likert-type scale with values ranging from 1 = Almost always/always happens to me to 5 = Never or rarely happens to me. The reliability indices obtained using Cronbach's α-statistic were: α superficial learning = 0.790, α deep learning = 0.773.

#### 2.2.2 Planning and decision-making

To analyze planning and decision making, the “Scale for the assessment of planning and decision making” (Darden et al., 1996) was used. The factor structure consists of a single factor including eight items (e.g., < < I think a lot in my head and analyze everything when I try to solve a problem>>). Responses are scored on a Likert-type scale ranging from 1 = Strongly Disagree to 7 = Strongly Agree. The reliability index obtained using Cronbach's α statistic was α = 0.818.

#### 2.2.3 Procrastination

The “Academic procrastination scala-short form” (APS-SF) psychometrically validated by Yockey (2016) was used to assess participants' level of procrastination. The factor structure of this version is a single factor composed of five items (e.g., < < I leave homework or class assignments to the last minute>>). Responses are scored on a Likert-type scale with values ranging from 1 = Completely false to 5 = Completely true. The reliability index obtained in this dimension was carried out using Cronbach's α statistic and has a result of α = 0.839.

### 2.3 Predictor/independent variables

#### 2.3.1 Bullying and cyberbullying

The level of bullying was assessed using the instrument “European bullying intervention project questionnaire” Spanish version of Ortega-Ruiz et al. (2016), 14 items, distributed in two dimensions: victimization (seven items) and aggression (seven items). Examples of items include: “Someone has hit or kicked me” and “I have insulted another colleague”. On the other hand, the Spanish version of the “European cyberbullying intervention project questionnaire” (ECIPQ; Del Rey et al., 2015) was used to assess cyberbullying, this instrument includes 22 items also divided into two dimensions: cyber-victimization (11 items) and cyber-aggression (11 items). Examples of items are: “Someone has spread rumors about me through the Internet” and “I have impersonated someone on social networks to make fun of him/her”. Reliability results are high for both bullying (Crombach's α victimization = 0.840 and Crombach's α aggression = 0.814) and cyberbullying (α cybervictimization = 0.872 and α cyberaggression = 0.877). Both questionnaires were administered individually and employ a Likert-type scale with a score ranging from 1 = Never to 5 = More than once a week. The items explore the frequency with which the described behaviors have occurred during the last 2 months and both require ~15 min to complete.

### 2.4 Confounding variables

#### 2.4.1 Age, body mass index, mother's education, and weekly physical activity

The age and educational level of each participant's mother were recorded using a socio-demographic data questionnaire (Baharvand et al., 2021; Zaatari and El Maalouf, 2022). Moreover, BMI (Bacon and Lord, 2021) was calculated using the Quetelet formula: weight (kg)/height^2^ (m). A digital scale ASIMED^®^ type B, class III and a portable measuring rod SECA^®^ 214 (SECA Ltd., Hamburg, Germany) were used to obtain weight and height measurements. Both measurements were taken in light clothing and without shoes. Weekly physical activity level was assessed using the “PACE+ Adolescent physical activity measure physical” questionnaire (Prochaska et al., 2001). This consists of two items asking the number of days on which the participants have performed at least 60 min of physical activity at moderate or vigorous intensity during the last 7 days and during a typical week. The final score was obtained by averaging both responses: (P1 + P2)/2). Its reliability index was α = 0.739.

### 2.5 Procedure

Data recording was carried out during the academic year 2022/23. A verbal and written description of the nature and purpose of the study was given to students, parents and legal guardians. Permission was also obtained from the school management and physical education teachers. The names of the participating students were coded to ensure anonymity and confidentiality. Each student completed questionnaires on bullying, attitudes toward studying, planning and decision-making, procrastination and a socio-demographic information table. During the completion of the questionnaires and the weight and height measurements, a specialized researcher gave instructions and monitored the time, while two research assistants observed possible doubts and any possible disturbances (e.g., space separation to ensure confidentiality of answers, noise outside the classroom, confused students, operation of electronic tools or internet connection). The study was approved by the Bioethics Commission of the University of Jaén (Spain), reference NOV.22/2.PRY. The design took into account the current Spanish legal regulations governing clinical research in humans (Royal Decree 561/1993 on clinical trials), as well as the fundamental principles established in the Declaration of Helsinki (2013, Brazil).

### 2.6 Statistical analysis

Comparison of continuous and categorical variables for all students and between boys and girls was carried out using Student's *t*-tests and χ2 tests, respectively. The normality and homoscedasticity of the data were verified using the Kolmogorov-Smirnov and Levene tests, respectively. To study whether adolescents with high levels of bullying and cyberbullying victimization/aggression had worse learning levels than those participants with low levels, an analysis of covariance (ANCOVA) was performed. Deep learning, superficial learning, planning and decision making, and procrastination were used as dependent variable and bullying victimization, bullying aggression, cyberbullying victimization, and cyberbullying aggression were entered as fixed factor. The bullying and cyberbullying values were dichotomized so that participants who stated that they had never been a victim/offender of bullying and/or cyberbullying (questionnaire score = 1) were labeled as “Never” and those who had ever been a victim/offender (questionnaire score = 2–5) were labeled as “Sometimes.” Because many comparison groups had different sample sizes, effect sizes were calculated using Hedges' g, where 0.2 = small effect, 0.5 = medium effect, and 0.8 = large effect (Martínez-López et al., 2018). The percentage of difference between groups (victims/non-victims - aggressors/non-aggressors)was calculated as: [(Large-measurement - small-measurement)/small-measurement] × 100. To find out the level of risk of bullying victimization/aggression and cyberbullying toward lower values in deep learning, superficial learning, planning and decision-making, and procrastination, a binary logistic regression was carried out. For this, the dependent variables were dichotomized by taking the median as a reference (Kobel et al., 2022; Zhong et al., 2023). Each strategy was classified as High ≥ median (reference group) vs. Low < median (risk group). In all analyses, age, BMI, mother's educational attainment and weekly physical activity were used as covariates. All analyses were conducted separately for boys and girls. A 95% confidence level (*p* < 0.05) was used for all results. All calculations were performed with the statistical software SPSS, v. 25.0 for WINDOWS (SPSS Inc., Chicago).

## 3 Results

### 3.1 Analysis of covariance of bullying and cyberbullying victimization with respect to deep and superficial learning, planning and school procrastination

**Figure 1:**
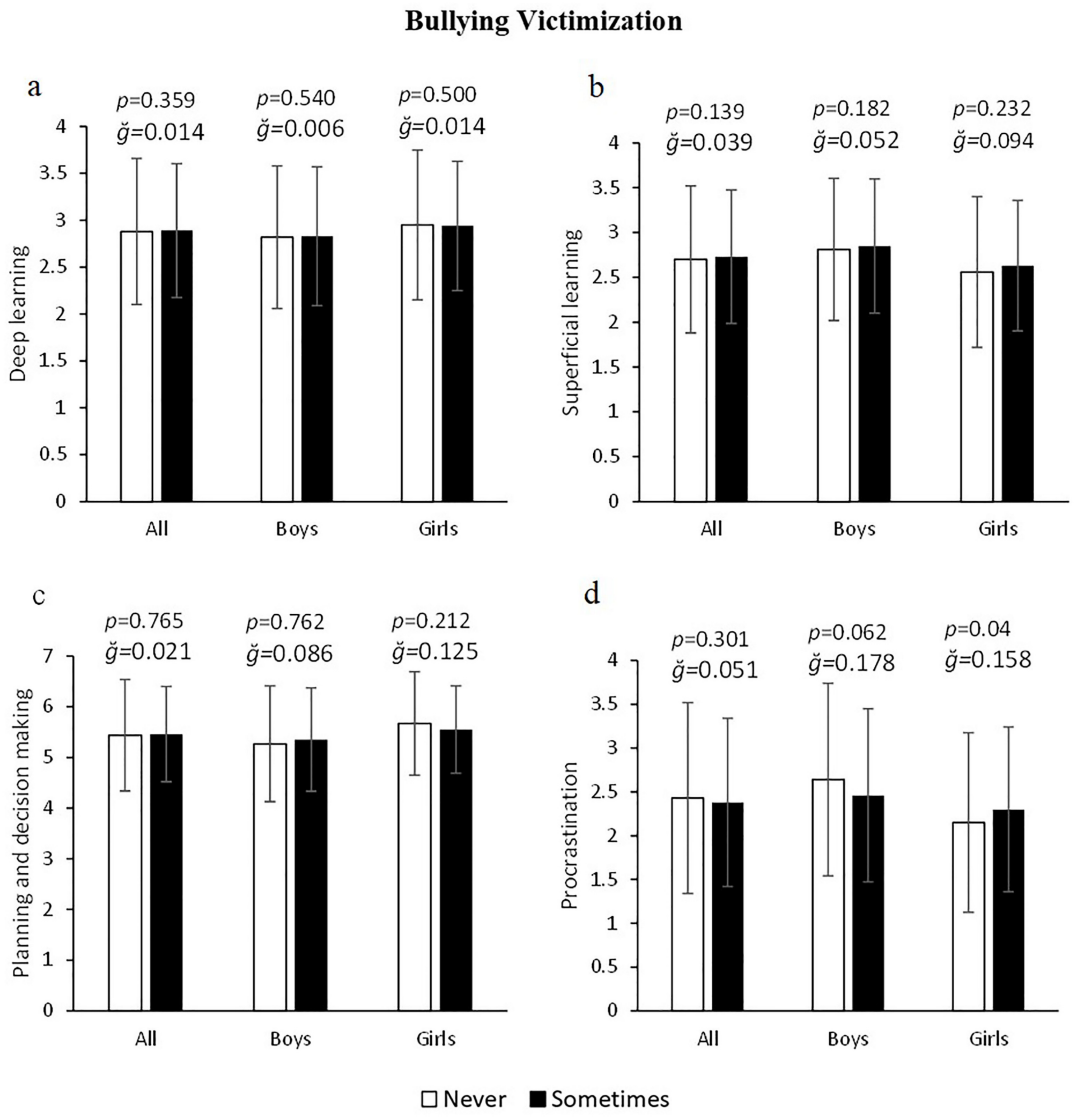
Association of bullying victimization with deep and superficial learning, planning and school procrastination.

**Figure 2:**
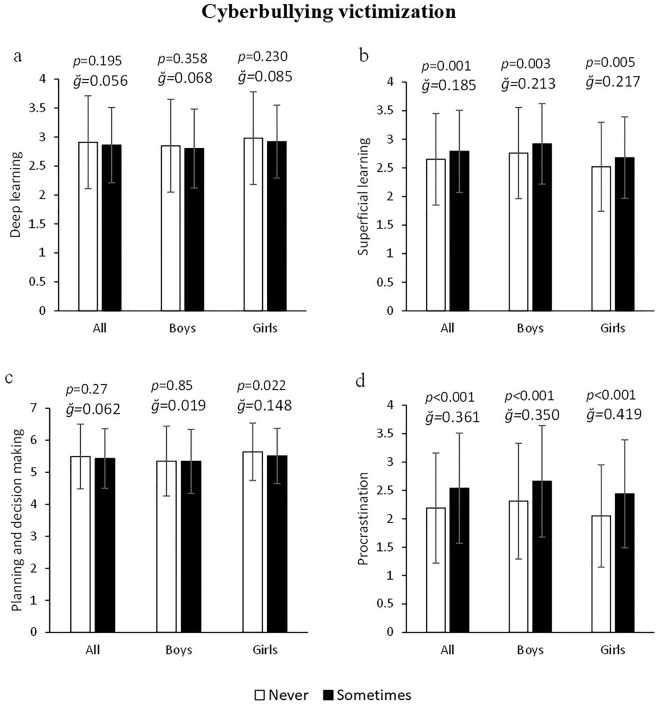
Association of cyberbullying victimization with deep and superficial learning, planning and school procrastination.

Overall, no significant differences were found in any learning variable as a function of bullying victimization (all *p* > 0.05; Figures 1a–d). Only, girls victimized by bullying showed higher procrastination toward class tasks (6.97%) than non-victimized girls (2.15 ± 1.03 vs. 2.3 ± 0.94 a.u.) F_(1, 643)_ = 4.241, *p* = 0.04, g = 0.158, 1–β = 0.538 (Figure 1d). On the other hand, both all young victims of cyberbullying and segmented by sex showed higher values in superficial learning: 5.28% (2.65 ± 0.8 vs. 2.79 ± 0.72 a.u.) F_(1, 1245)_ = 12.102, *p* = 0.001, g = 0.185, 1–β = 0.935 (Figure 2b) and procrastination: 15.98% (2.19 ± 0.97 vs. 2.54 ± 0.97 a.u.) F_(1, 1245)_ = 45.275, *p* < 0.001, g = 0.361, 1–β = 0.998 (Figure 2d). In turn, the data also showed that planning and decision making was significantly lower in cyberbullying victimized girls: 2.36% (*p* = 0.022) but not in victimized boys (*p* > 0.05), Figure 2c. No significant differences in deep learning were found as a function of cyberbullying victimization in either boys or girls (all *p* > 0.05, Figure 2a).

### 3.2 Analysis of covariance of aggression in bullying and cyberbullying with respect to deep and superficial learning, planning and school procrastination

**Figure 3:**
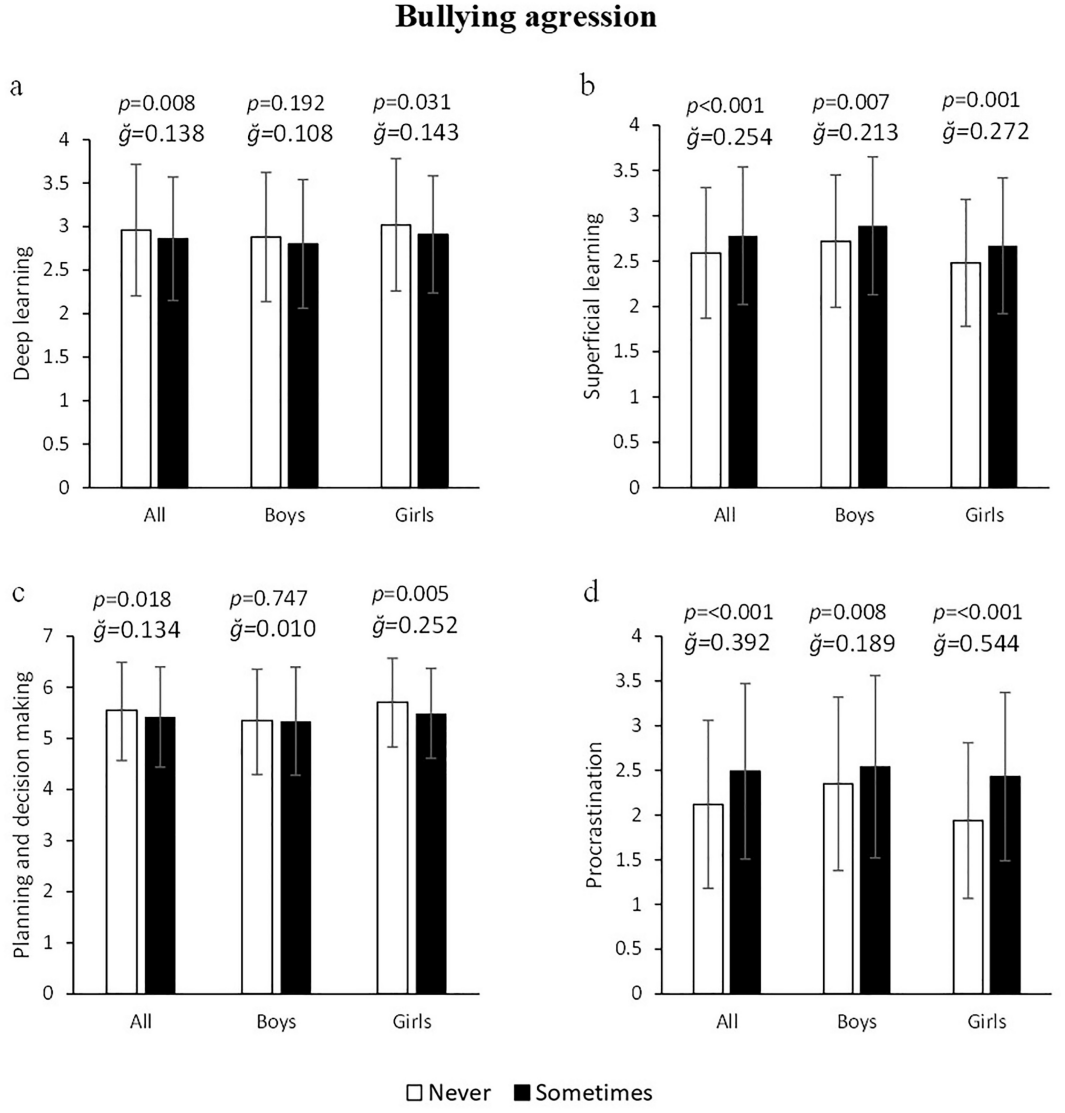
Association of bullying agression with deep and superficial learning, planning and school procrastination.

Young bullying aggressors showed significantly lower deep learning: −3.5% (2.96 ± 0.76 vs. 2.86 ± 0.71 a.u.) F_(1, 1245)_ = 6.977, *p* = 0.008, g = 0.138, 1–β = 0.752 (Figure 2a), higher superficial learning: 7.34% (2.59 ± 0.72 vs. 2.78 ± 0.76 a.u.) F_(1, 1245)_ = 20.673, *p* < 0.001, g = 0.254, 1–β = 0.995 (Figure 2b), lower values in planning and decision making: −2.40% (5.55 ± 0.94 vs. 5.42 ± 0.98 a.u.) F_(1, 1245)_ = 5.614, *p* = 0.018, g = 0.134, 1–β = 0.658 (**Fiureg 2c**) and high procrastination: 17.45% (2.12 ± 0.94 vs. 2.49 ± 0.98 a.u.) F_(1, 1245)_ = 48.346, *p* < 0.001, g = 0.392, 1–β = 1.000, compared to non-aggressors. More specifically, aggressor girls had worse values in all learning variables: Deep learning: −3.78% (*p* = 0.031, Figure 3a), superficial learning: 7.66% (*p* = 0.001, Figure 3b), planning: −4.01% (*p* = 0.005, Figure 3c) and procrastination: 25.26% (*p* < 0.001, Figure 3d). In boys, significant differences were only found in superficial learning and procrastination, where offenders showed higher values: 6.25% (*p* = 0.007, Figure 3b) and 8.09% (*p* = 0.008, Figure 3c), respectively.

**Figure 4:**
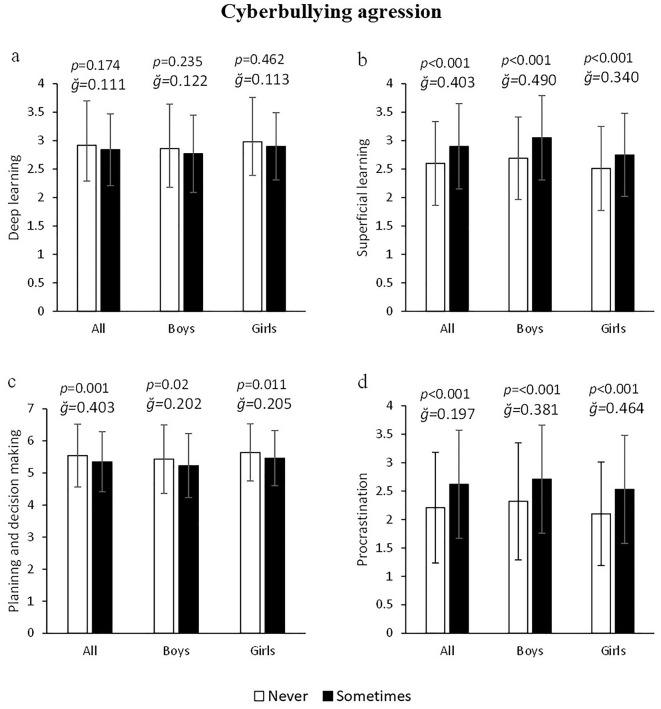
Association of cyberbullying agression with deep and superficial learning, planning and school procrastination.

Similarly, cyberbullying aggressors had higher values of superficial learning: −11.54% (2.6 ± 0.74 vs. 2.9 ± 0.75 a.u.) F_(1, 1245)_ = 44.154, *p* < 0.001, g = 0.403, 1–β = 1. 000 (Figure 4b) and procrastination toward class tasks: 18.55% (2.21 ± 0.97 vs. 2.62 ± 0.95) F_(1, 1245)_ = 44.362, *p* < 0.001, g = 0.197, 1–β = 1.000 (Figure 4d), as well as lower planning: −3.55% (5.54 ± 0.98 vs. 5.35 ± 0.94 a.u.) F_(1, 1245)_ = 11.477, *p* = 0.001, g = 0.403, 1–β = 0.923, than those who are not cyberbullying aggressors (Figure 4c). Sex segmented analysis indicated that, in both boys and girls, cyberbullying offenders had higher values of superficial learning (boys =13.38% and girls = 9.56%; both *p* < 0.001, Figure 4b), lower planning scores (boys = 3.82% and girls = 3.3%; both *p* < 0.020, Figure 4c) and higher procrastination toward class tasks (boys = 16.81% and girls = 20.48%; both *p* < 0.001, Figure 4d). No statistically significant differences were found overall, nor segmented by sex, in the deep learning variable as a function of cyberbullying aggression (all *p* > 0.05) (Figure 4a).

### 3.3 Binary logistic regression on bullying and cyberbullying victimization and aggression with respect to deep and superficial learning, planning and school procrastination

Data showing the risk of exposure to bullying and cyberbullying victimization/aggression with respect to the learning variables are shown in Table 2. Victims of bullying were 1.5 and 1.9 times more likely, and thus at greater risk, than non-victims to have high values for superficial learning [Odds ratio(OR) = 1.500; *p* < 0.001] and procrastination toward class tasks (OR = 1.910, *p* < 0.001), respectively. Similar risk values were obtained for the two previous variables when the analysis was carried out separately for boys and girls (both OR >1.47; *p* < 0.001). On the other hand, cyberbullying victims were 3.6, 1.4, and 4.3 times more likely than non-victims to have high values of superficial learning, low planning and high procrastination (OR = 3.579, OR = 1.444, and OR = 4.335, respectively, all *p* < 0.008). Sex-differentiated results showed that boys and girls victims of cyberbullying had a similar risk of superficial learning (both OR > 3.6; *p* < 0.001) and procrastination (both OR > 4.2; *p* < 0.001). However, within cyberbullying victimization, the risk of poor planning was only significant in girls (OR =1.730, *p* = 0.006). Finally, the risk probability of low deep learning outcomes was not significant in any case (all *p* > 0.05).

**Table 2:** OR and 95% CI for levels of victimization/aggression in bullying and cyberbullying according to learning indicators in schoolchildren and adolescents.

		**All (1,251)**				**Boys (602)**				**Girls (649)**			
		***N***	***p***	**OR**	**95% CI**	***N***	***p***	**OR**	**95% CI**	***N***	***p***	**OR**	**95% CI**
**Bullying victimization**													
Deep learning	High	606		1	Referent	260		1	Referent	346		1	Referent
	Low	645	0.364	1.073	0.922–1.248	342	0.140	1.177	0.948–1.463	303	0.902	0.986	0.793–1.227
Superficial learning	Low	628		1	Referent	262		1	Referent	366			Referent
	High	623	< 0.001	1.50	1.28–1.747	340	< 0.001	1.476	1.180–1.846	283	0.001	1.561	1.248–1.954
Planning and decision making	High	600		1	Referent	264		1	Referent	336		1	Referent
	Low	651	0.536	1.049	0.902–1.218	338	0.176	1.160	0.936–1.437	313	0.678	0.955	0.770–1.185
Procrastination	Low	588		1	Referent	254		1	Referent	334			Referent
	High	663	< 0.001	1.91	1.611–2.265	348	< 0.001	1.59	1.261–2.013	315	< 0.001	2.396	1.858–3.091
**Bullying aggressor**													
Deep learning	High	606		1	Referent	260		1	Referent	346			Referent
	Low	645	0.079	1.101	0.969–1.582	342	0.147	1.236	0.928–1.647	303	0.097	1.263	0.958–1.664
Superficial learning	Low	628		1	Referent	262		1	Referent	366			Referent
	High	623	< 0.001	2.88	2.256–3.668	340	< 0.001	2.524	1.807–3.526	283	< 0.001	3.167	2.214–4.531
Planning and decision making	High	600		1	Referent	264		1	Referent	336			Referent
	Low	651	0.004	1.331	1.094–1.620	338	0.175	1.211	0.918–1.596	313	0.024	1.386	1.044–1.840
Procrastination	Low	588		1	Referent	254		1	Referent	334			Referent
	High	663	< 0.001	3.468	2.650–4.538	348	< 0.001	2.793	1.951–4.00	315	< 0.001	4.213	2.789–6.365
**Cyberbullying victimization**													
Deep learning	High	606		1	Referent	260		1	Referent	346			Referent
	Low	645	0.216	0.847	0.650–1.102	342	0.074	0.697	0.470–1.035	303	0.985	0.996	0.691–1.437
Superficial learning	High	628		1	Referent	262		1	Referent	366			Referent
	Low	623	< 0.001	3.579	2.498–5.128	340	< 0.001	3.943	2.179–6.778	283	< 0.001	3.557	2.217–5.707
Planning and decision making	High	600		1	Referent	264		1	Referent	336			Referent
	Low	651	0.008	1.444	1.100–1.895	338	0.315	1.213	0.832–1.767	313	0.006	1.730	1.166–2.568
Procrastination	Low	588		1	Referent	254		1		334			Referent
	High	663	< 0.001	4.335	2.927–6.422	348	< 0.001	4.768	2.587–8.787	315	< 0.001	4.192	2.489–7.060
**Cyberbullying aggressor**													
Deep learning	High	606		1	Referent	260		1	Referent	346			Referent
	Low	645	0.532	0.914	0.688–1.213	342	0.067	0.668	0.433–1.029	303	0.557	1.115	0.761–1.634
Superficial learning	Low	628		1	Referent	262		1	Referent	366			Referent
	High	623	< 0.001	7.636	4.460–13.073	340	< 0.001	9.636	4.181–22.203	283	< 0.001	6.174	3.061–12.453
Planning and decision making	High	600		1	Referent	264				336			Referent
	Low	651	< 0.001	2.270	1.601–3.217	338	< 0.010	1.886	1.164–3.056	313	< 0.001	2.624	1.573–4.374
Procrastination	Low	588		1	Referent	254		1	Referent	334			Referent
	High	663	< 0.001	10.068	5.502–18.421	348	< 0.001	12.226	4.935–30.288	315	< 0.001	< 0.0018.346	3.689–18.885

Deep learning, superficial learning, planning and procrastination were included in the logistic regression as a categorical variable (low vs. high). OR was adjusted for age, body mass index, mother's educational level and average weekly physical activity. OR, odds ratio; CI, confidence interval.

On the other hand, bullying aggressors were 2.9, 1.3, and 3.5 times more likely than non-aggressors to have high values of superficial learning, low planning and high procrastination (OR = 2.880, OR = 1.331 and OR = 3.468, respectively, all *p* < 0.004). Sex-differential analysis showed significant risk scores in aggressor girls but not in boys (superficial learning: OR = 3.167, *p* < 0.001; planning and decision making: OR = 1.386, *p* = 0.024 and procrastination: OR = 4.213, *p* < 0.001). In turn, cyberbullying aggressors were 7.6, 2.3, and 10.1 times more at risk than non-aggressors for high values of superficial learning, low planning and high procrastination (OR = 7.636, OR = 2.270, and OR = 10.068, respectively, all *p* < 0.001). Similar risk values were obtained for the above three variables when the analysis was conducted separately for boys and girls (all *p* < 0.001). Finally, the risk probability of low deep learning outcomes was not significant in any case (all *p* > 0.05).

## 4 Discussion

The aim of the present study was to analyze the association of bullying and cyberbullying victimization and aggression with different learning variables in schoolchildren and adolescents of both sexes, compared to those not involved in bullying and cyberbullying. In general, the results have shown that both victims and aggressors of bullying and cyberbullying present a more superficial learning, have lower values in planning and decision making and tend to delay class tasks than those not involved in acts of bullying and cyberbullying. Girls who are victims of bullying are affected more than boys and are twice as likely to procrastinate as non-victims. The data also show that cyberbullying is more negatively associated with learning variables than traditional bullying. This risk is multiplied × 3 and × 4 toward having high values of superficial learning and procrastination, respectively. In the case of cyberbullying, the negative effects suffered by aggressors on learning variables are even more pronounced. In both sexes, the risk of exposure to having high values of superficial learning, low planning and high procrastination is multiplied × 8, × 2, and × 10, respectively.

According to the present study, young people affected by bullying and cyberbullying, regardless of being a victim or aggressor, are not associated with any positive effect on the studied variables of deep learning, superficial learning, planning and procrastination toward class tasks. These findings coincide with Bergmann (2022), who found no significant associations between bullying and positive effects on student learning variables. Likewise, the results obtained in this work, coincide with previous research that attributed to students affected by bullying lower academic grades and a predominance of superficial learning (De Aroni and Corcuera, 2021; Rigby, 2003; Ruiz-Piñero and Ramírez-Cerezo, 2011), more time to plan and make decisions (Rivera, 2018) and greater academic procrastination (Hamidipour and Ezadian, 2023; Nwosu et al., 2020).

On the other hand, the negative effects of bullying and cyberbullying on learning are more harmful in girls than in boys. According to these findings, several research studies that associated bullying/cyberbullying with anxiety or depression variables converge in that girls are more susceptible to the negative consequences of bullying than boys (Carvalho et al., 2021; Eyuboglu et al., 2021; Islam et al., 2022). Within the educational field, recent studies have associated bullying behaviors with learning variables, with girls obtaining worse academic results compared to boys (Riffle et al., 2021; Halliday et al., 2021). Specifically, this study found that aggressor girls had worse values on all learning variables (deep learning, surface learning, planning and procrastination). According to this finding, Riffle et al. (2021) in his research study, associated bullying with grades, with the result that girls involved in bullying showed negative associations in their academic performance compared to boys. In contrast, another study that associated bullying with academic performance level did not show significant differences in terms of sex, but rather that variations in learning variables depended more on the role associated with the student (Obregón-Cuesta et al., 2022).

However, for other researchers, victims of bullying or cyberbullying obtained lower academic performance than non-victims, with no significant differences between boys and girls (Kim et al., 2020). The present study coincides with the current trend aimed at addressing bullying and cyberbullying as public health problems, with a special focus on protecting and supporting girls. The main value of the present research lies in providing quantitative results with risk calculation aimed at assessing the effect on learning variables, especially in girls, who seem to be at higher risk of suffering the adverse consequences of bullying and cyberbullying.

Another aspect that generates controversy is the possible differentiating role of victimization and aggression on learning variables. Our data have revealed that, similar to victims, young aggressors also present low values in most of the learning variables. For example, bullying aggressor girls showed lower values in deep learning, as well as in planning and decision making, in addition to acquiring more superficial learning and tending to procrastinate on class tasks. In line with these results, recent research has shown that the bullying climate, whether victim or aggressor is directly associated with student learning (Delprato et al., 2017; Huang, 2022). Specifically, several studies have found that bullying students have lower levels of deep learning and higher levels of superficial learning (AlBuhairan et al., 2017), low planning and responsible decision making (Llorent et al., 2021) and high procrastination (Rebetez et al., 2018).

Our data also revealed that both victimization and aggression, derived from cyberbullying, have a stronger negative association with learning variables than traditional bullying. Similarly, recent researchers have suggested that both victimization and aggression in cyberbullying turn out to be more harmful on learning variables compared to traditional bullying (Alotaibi, 2019; Graham, 2023; John et al., 2023). It appears that cyberbullying, due to its pervasive and anonymous nature, has a more profound and lasting impact on learning (Graham, 2023), negative consequences on students' academic performance (Alotaibi, 2019) and significant impact on emotional and mental wellbeing (John et al., 2023). However, other works argue that cyberbullying and traditional bullying negatively affect psychological symptoms and wellbeing, without clearly differentiating which of the two has a more severe impact on learning (Carvalho et al., 2021; Kim et al., 2022). Based on the information provided, a clear general trend is shown indicating that cyberbullying may have more severe consequences due to its invasive and persistent nature.

### 4.1 Limitations and strengths

The present study has some methodological and procedural limitations that should be pointed out. Among them, those inherent to the cross-sectional nature of the design, which does not allow establishing causal relationships and depends on the veracity with which the participants respond to the different measures applied. There is a possibility that some students may have provided responses oriented toward maintaining a positive self-image. Another limitation of the present study was that the sample was recruited exclusively in a specific region of Spain, which could limit the generalizability of the results to other geographical and sociocultural contexts. In addition, the sample was selected by convenience, which prevents it from being representative of the Spanish population. On the other hand, among the measures that underpin the robustness of our data are the use of coding techniques that guarantee the anonymity and confidentiality of the participants, the application of instruments of high reliability and proven internal validity, as well as rigorous data collection and exhaustive compliance with the procedure. Finally, the use of a large number of covariates (age, BMI, mother's educational level and average weekly physical activity) shows unprecedented results in the field of education.

## 5 Conclusions and practical applications

The present study allows us to conclude that boys and girls who are victims of bullying have almost twice the risk of having higher values of superficial learning and procrastination toward class tasks than non-victims. In the case of girls, procrastination toward class tasks increases by 7%. Victims of cyberbullying present 5.3% more superficial learning and 16% more procrastination toward class tasks than non-victims, multiplying the risk due to this exposure by 3 and 4, respectively. In general, bullying and cyberbullying aggressors have more negative learning values than non-aggressors. Young cyberbullying aggressors register lower values of deep learning (−11.5%) and acquire more superficial learning (boys = 13.4% and girls = 9.6%), plan worse (boys = 3.8% and girls = 3.3%) and tend to procrastinate on class tasks (boys = 16.8% and girls = 20.5%). In both sexes, the risk of exposure to having high values of superficial learning, low planning and high procrastination is multiplied × 8, × 2, and × 10, respectively.

This study underscores the need to develop specific intervention strategies to mitigate the effects of bullying and cyberbullying on learning. The identification of an increased risk of superficial learning, low planning and procrastination in victims and aggressors not only reinforces the evidence on the consequences of bullying, but also points to the urgency of implementing prevention programs aimed at improving the quality of learning in affected students.

From an educational perspective, the results suggest that schools should adopt multidimensional approaches that integrate psycho-pedagogical interventions aimed at improving academic self-regulation and decision-making in the classroom. In turn, teacher training in early identification and management of bullying, as well as the promotion of active methodologies that foster deep learning and strategic planning in students, may be key tools to counteract the negative effects observed. At the scientific level, these findings contribute to the understanding of the phenomenon of bullying beyond its emotional and social repercussions. In addition, the inclusion of covariates such as body mass index, maternal educational level and weekly physical activity allows for a more precise analysis applicable to diverse educational contexts, providing a framework for future research seeking to deepen the relationship between bullying and academic performance.

## Data Availability

The raw data supporting the conclusions of this article will be made available by the authors, without undue reservation.
